# A rare case of male Fournier’s gangrene with mixed *Actinomyces turicensis* infection

**DOI:** 10.1186/s12894-022-00975-z

**Published:** 2022-02-23

**Authors:** Tong-chun Mao, Xuan Zhou, Meng-nan Tian, Yi-ming Zhang, Shao-liang Wang

**Affiliations:** Department of Plastic and Cosmetic Surgery, Xinqiao Hospital, Army Medical University, Chongqing, China

**Keywords:** Fournier's gangrene, *Actinomyces*, Necrotizing fasciitis, Skin grafting, Case report

## Abstract

**Background:**

Fournier's gangrene (FG), a urological emergency with high mortality, is an infectious necrotizing fasciitis of the perineal and genital regions. The majority of FG is caused by polymicrobial organisms involving mixed aerobes and anaerobes but rarely reveals *Actinomyces* species.

**Case presentation:**

We report a healthy 67-year-old Asian male who presented with rapidly progressive painful swelling of the scrotum. Clinically diagnosed with FG, the patient underwent an emergency radical debridement, followed by broad-spectrum antibiotics and negative pressure wound therapy. The identification of the causative microorganisms showed *Actinomyces turicensis* and the antibiotic treatment was adjusted accordingly. After wound bed preparation, we took split-thickness skin grafts to cover the scrotal wound. Active management to minimize faecal contamination was applied throughout the whole course of treatment and repair. The patient was satisfied with the outcome. This was an extremely rare case of *A. turicensis* as the main causative pathogen of FG*.*

**Conclusions:**

FG due to *Actinomyces* species is rarely reported, but we should still consider this pathogenic microorganism that has long been neglected.

## Background

Fournier’s gangrene (FG), a type of necrotizing fasciitis, is a rare but fatal disease involving the perineal and genital regions. It mostly occurs in elderly males with a mortality rate as high as 40% [1]. The scrotum and penis but not the testicles are often involved due to the autonomy of their blood supply [2]. FG is often induced by breach of the urethral or gastrointestinal mucosa [3]. The infection initially spreads in the fascia and does not invade the overlying soft tissue and thus only presents as local swelling, fever, and tenderness. Then bacterial spread causes obliterative endarteritis, resulting in aggressive ischaemic necrosis of the fascia, and the affected areas turn into ulceration skin gangrene with sensory loss after 3–8 days, or even bacteraemia and septic shock in severe cases [4]. The most common pathogen of FG is polymicrobial organisms involving mixed aerobes and anaerobes, followed by *Escherichia coli* and *Streptococcus* [5, 6]. Here, we report a rare case of FG mixed with *A. turicensis* infection.

## Case presentation

**Fig. 1:**
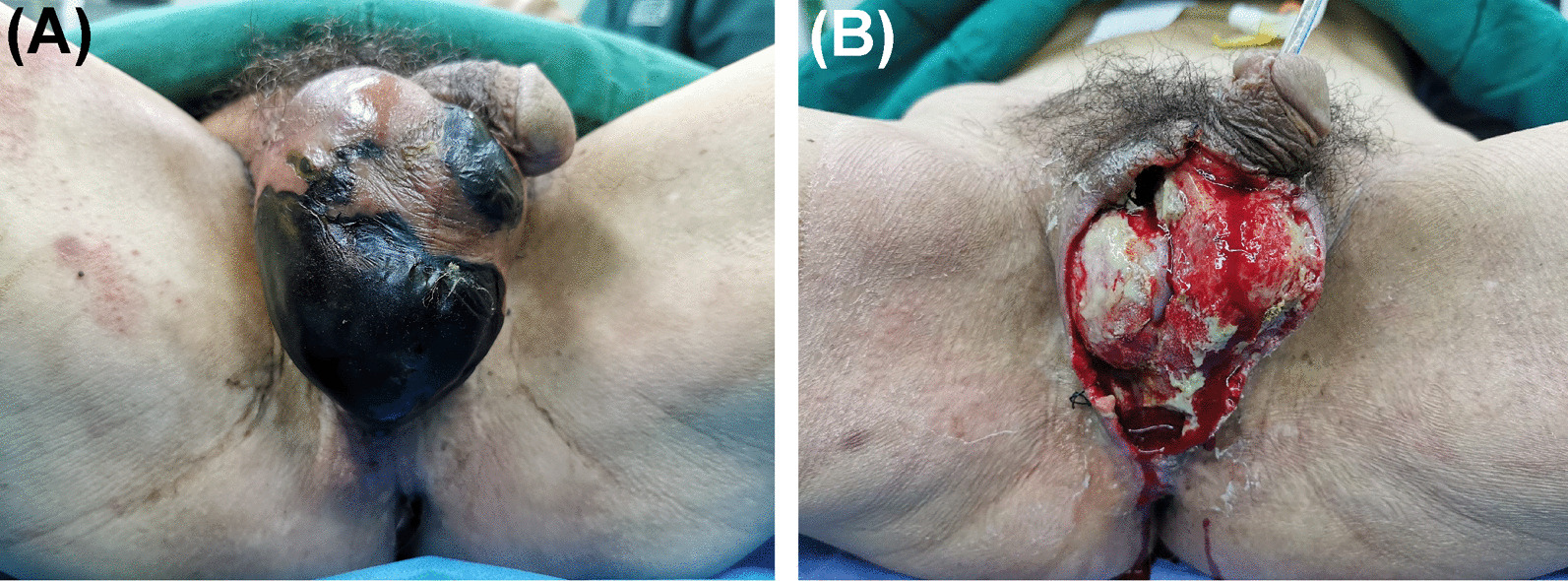
Representative images of gangrenous perineum. **a** Swelling scrotum with gross skin necrosis, stench and oedema. Image taken before 1st debridement. **b** The total scrotal skin defect covered by purulent necrotizing tissues. Image taken before 2nd debridement when the negative pressure dressing was just removed

An afebrile 67-year-old Asian male without systemic diseases presented to the emergency department with painful swelling in the scrotum (Fig. 1a). He reported developing redness and swelling around the scrotum after painful defecation 5 days before admission, and his symptoms worsened 2 days prior, with extensive scrotal skin necrosis, stench and severe pain. There was no history of diabetes mellitus or urethral trauma. The biochemistry results showed a white blood cell count of 24.14 × 10^9^/L (neutrophils 92.2%, lymphocytes 2.3%, monocytes 5.5%) and a C-reactive protein level of 122.0 mg/L.

After the clinical diagnosis of FG was made, the patient underwent an emergency radical debridement. During the operation, we found intact sheaths of his testicles, an intact anal sphincter, and a 15 cm sinus along the spermatic cord in the right inguinal area, with no visible sulphur granules within the wound and no subcutaneous crepitus. Postoperatively, he was treated with cefperazone-sulbactam for anti-infection, negative pressure wound therapy (NPWT), urinary catheterization, and an individualized diet. Pathological analysis revealed acute and chronic inflammation in the scrotum, with focal necrosis. Cultures of the exudates from the scrotal wound grew *A. turicensis* while no pathogens in urine culture were found; thus, the antibiotics were changed to piperacillin-sulbactam and clindamycin. For wound bed preparation of skin grafting, we gave the patient three debridements followed by NPWT (Fig. 1b). Then, split-thickness skin grafts approximately 0.3 mm thick were taken from his left thigh to cover the wound.

**Fig. 2:**
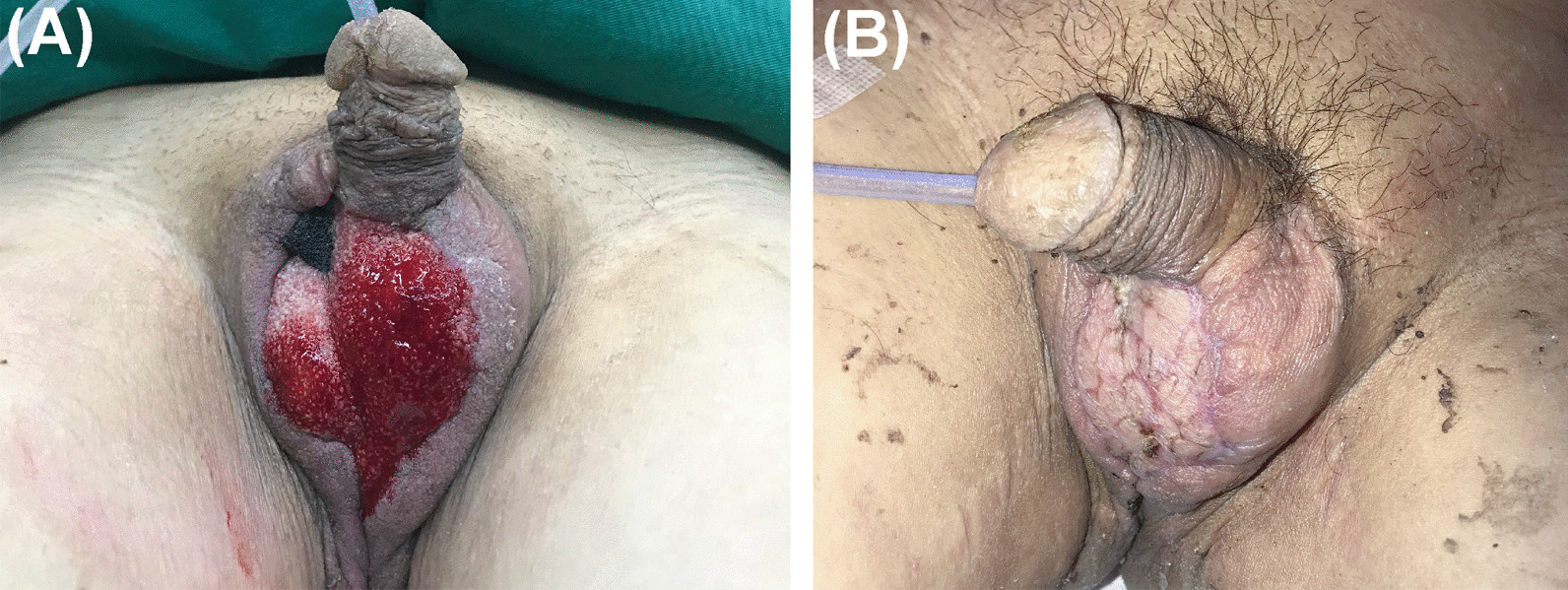
Representative images of perineum without infection. **a** Granulation tissue covered the scrotal wound and the defect size decreased due to using NPWT. Image taken before skin grafting. **b** Complete wound closure. Image taken 14 days after skin grafting

Two weeks after skin grafting (Fig. 2b), graft take was 95%. The patient was satisfied with the outcome and discharged. No relapse of the infection was observed during the 3-month follow-up.

## Discussion and conclusions

FG is a fulminant infection with extremely high mortality. Once diagnosed with FG, prompt extensive debridements and broad-spectrum antibiotics are two key points [4]. After systemic treatment of the infection, reconstructive techniques should be implemented in time to close or cover the defects of the perineal and genital regions.

Wound cultures of FG often shows polymicrobial infection of both aerobes and anaerobes but rarely reveal *Actinomyces* species [7]. As a facultative Gram-positive anaerobe, *Actinomyces* are typically found in the human genitourinary tract (*A. turicensis* commonly isolated from [8]), digestive tract and oral cavity, mostly causing chronic purulent inflammation [9]. This chronic and recurrent abscess formation is related to the lack of obvious clinical manifestations in the early acute phase of actinomycotic infection, the rigorous culture conditions of *Actinomyces* species, or a failure to timely and correctly diagnose and treat of the infection. In this case, the infection was taken seriously in the acute stage due to the rapid deterioration of the patient’s genital condition. Generally, actinomycotic infection is not specific in symptoms and signs and sulphur granules in the pus are considered to be a prominent clinical diagnostic feature [9]. However, in this case with *A. turicensis* infection, no sulfur granules were found [10]. In fact, the formation of the granules depends on the growth environment and the morphology of *Actinomyces*. Therefore, sulphur granules do not always exist. Microbiologic culture and identification are the gold standard for detecting actinomycotic lesions.

In treatment, given the patient’s relatively good general condition at admission, we gave him radical and repeated debridement and broad-spectrum antibiotics in time. Regarding the choice of antibiotics, although the result of the first germiculture only suggested *Actinomyces* as the causative organism, it is known that most actinomycotic infections are polymicrobial [9], because some concomitant aerobes (such as *Escherichia coli*, *Streptococcus* and *Staphylococcus*) consume oxygen to make the environment more conducive to the growth and proliferation of *Actinomyces* and strict anaerobes. Combined with the pathogenic features of *A. turicensis* and the relevant clinical history (the onset of his symptoms following forced defecation) and symptoms of the patient, we considered that the patient suffered from a polymicrobial enterogenic infection. Accounting for the fulminant progress and high level of mortality of FG [1, 4] and the possible long period of identifying certain microbes, we immediately applied broad-spectrum antibiotics after the first debridement and later replaced the antimicrobial agents with those covering *A. turicensis*. Importantly, surveillance standards for *Actinomyces* are lacking in both the World Health Organization and developed countries as its importance as a pathogen has been neglected for a long time [11]. Without commercial kits, antimicrobial susceptibility testing of *Actinomyces* species is performed in only a few clinical microbiology laboratories [12], so empirical medication is widely accepted.

To effectively control the severe infection of FG, in addition to intravenous antibiotics, potent faecal management is compulsory during the entire treatment and repair process. There are many physiological folds in the skin around the perineum, and FG infection often involves the perianal area and affects the normal excretion of urinary or faecal material, leading to wounds of FG patients being continually exposed to a higher risk of contamination than those of many other infections. Apart from the fact that FG commonly develops in immunocompromised patients with comorbidities, such as diabetes mellitus and malignancy [4, 13], even for immunocompetent patients, immune functions may be greatly reduced under stress; thus, the normal microflora of the human gut or skin, such as *A. turicensis,* may transform into pathogens. For patients without sphincter injury, not using surgical faecal diversion not only avoid wound contamination [14] but also can improve the prognosis of FG [15] as well as the patients’ long-term quality of life. In this case, based on the necrotic extent of the patient’s perineum (with an intact anal sphincter and without defecation problems) and his preference, we chose NPWT instead of a preventive colostomy before skin grafting to accelerate the preparation of the wound bed without faecal contamination, and a clinical outcome was achieved. As one of the most important methods for acute or chronic wounds in recent times [16], NPWT is also of great significance for promoting FG healing. First, the airtight film dressing isolates the wound and prevents the invasion of bacteria in excrement. Second, continuous negative pressure suction removes necrotic tissue and exudates, reduces the bacterial load and avoids the absorption of harmful substances. Third, two impressive features of NPWT are to stimulate the formation of vascularized granulation and tissue and to decrease wound surface area (Fig. 2a), which subsequently reduces the preparation time for skin grafting. Meanwhile, to minimize the faecal volume and frequency and the risk of forced defecation at the same time to balance his willingness to consume oral feed, during the hospitalization period, the patient received personalized enteral nutrition, supplemented with a low-residue diet. Perineal cleansing was applied by the nurses three times a day.

Here, we describe a rare case of FG mainly caused by *A. turicensis*, which was effectively treated by early aggressive debridements, broad-spectrum antibiotics, together with split-thickness skin grafting and faecal management. Although necrotizing fasciitis such as FG mainly caused by *Actinomyces* species is seldom reported in either immunocompetent or immunocompromised patients, we should still consider it as a suspected causative agent and avoid missed diagnosis.

## Data Availability

Not applicable.

## Abbreviations

FG: Fournier's gangrene
*A. turicensis*: *Actinomyces turicensis*
NPWT: Negative pressure wound therapy

## References

[1] Yanar H, Taviloglu K, Ertekin C, Guloglu R, Zorba U, Cabioglu N (2006). Fournier’s gangrene: risk factors and strategies for management. World J Surg.

[2] Hagedorn JC, Wessells HA (2017). A contemporary update on Fournier’s gangrene. Rev Nat Rev Urol.

[3] Stevens DL, Bryant AE (2017). Necrotizing soft-tissue infections. N Engl J Med.

[4] Chernyadyev SA, Ufimtseva MA, Vishnevskaya IF, Bochkarev YM, Ushakov AA, Beresneva TA (2018). Fournier’s gangrene: literature review and clinical cases. Urol Int.

[5] Tang LM, Su YJ, Lai YC (2015). The evaluation of microbiology and prognosis of Fournier’s gangrene in past five years. Springerplus.

[6] Çalışkan S, Özsoy E, Sungur M, Gözdaş HT (2019). Fournier’s gangrene: Review of 36 cases. Ulus Travma Acil Cerrahi Derg.

[7] Tena D, Losa C, Medina-Pascual MJ, Sáez-Nieto JA (2014). Fournier’s gangrene caused by Actinomyces funkei, Fusobacterium gonidiaformans and Clostridium hathewayi. Anaerobe.

[8] Clarridge IIIJE, Zhang Q (2002). Genotypic diversity of clinical Actinomyces species: phenotype, source, and disease correlation among genospecies. J Clin Microbiol.

[9] Könönen E, Wade WG (2015). Actinomyces and related organisms in human infections. Clin Microbiol Rev.

[10] Pulverer G, Schütt⁃Gerowitt H, Schaal KP (2003). Human cervicofacial actinomycoses: microbiological data for 1997 cases. Clin Infect Dis.

[11] Urbán E, Gajdács M (2021). Microbiological and clinical aspects of Actinomyces infections: What have we learned?. Antibiot (Basel).

[12] Jenkins SG, Schuetz AN (2012). Current concepts in laboratory testing to guide antimicrobial therapy. Mayo Clin Proc.

[13] Akan S, Urkmez A (2020). Association between atherogenic dyslipidemia and Fournier’s gangrene. Rev Assoc Med Bras (1992).

[14] Goh M, Chew MH, Au-Yong PS, Ong CE, Tang CL (2014). Nonsurgical faecal diversion in the management of severe perianal sepsis: a retrospective evaluation of the flexible faecal management system. Singap Med J.

[15] Yeniyol CO, Suelozgen T, Arslan M, Ayder AR (2004). Fournier’s gangrene: experience with 25 patients and use of Fournier’s gangrene severity index score. Urology.

[16] Lalezari S, Lee CJ, Borovikova AA, Banyard DA, Paydar KZ, Wirth GA (2017). Deconstructing negative pressure wound therapy. Int Wound J.

